# Current Evidence on the Benefit of Exercise in Cancer Patients: Effects on Cardiovascular Mortality, Cardiotoxicity, and Quality of Life

**DOI:** 10.31083/j.rcm2406160

**Published:** 2023-06-06

**Authors:** Núria Coma, Sergio Moral, Esther Ballesteros, Arantxa Eraso, Montse Ventura, Elisabet Pujol, Ramon Brugada

**Affiliations:** ^1^Cardiology Department, Hospital Universitari Doctor Josep Trueta and Hospital Santa Caterina, 17007 Girona, Spain; ^2^Medical Science Department, School of Medicine, University of Girona, 17003 Girona, Spain; ^3^Centro de Investigación Biomédica en Red de Enfermedades Cardiovasculares (CIBERCV), 28029 Madrid, Spain; ^4^Dirección Territorial de Radiologia y Medicina Nuclear de Girona, Insititut de Diagnòstic per la Imatge (IDI), 17007 Girona, Spain; ^5^Radiation Oncology Department, Institut Català d’Oncologia, 17007 Girona, Spain; ^6^Radiation Oncology Department, Institut Català d’Oncologia, L'Hospitalet de Llobregat, 08908 Barcelona, Spain; ^7^Center for Cardiovascular Genetics, Biomedical Research Institute of Girona, 17190 Girona, Spain

**Keywords:** cardiotoxicity, cardiorespiratory fitness, cardio-oncology, cardiovascular health

## Abstract

Cancer and its treatments affect cardiovascular (CV) health, including an increased risk of CV death, decreased cardiorespiratory fitness (CRF), and cardiac dysfunction. Moreover, cancer-related fatigue and worse quality of life (QoL) are highly prevalent adverse effects experienced by patients during treatment and can persist years after therapy ends. Physical exercise has been proposed as a strategy to improve different aspects of life of cancer patients, and is recommended as a therapy in cardio-oncology guidelines. Exercise interventions reduce fatigue and improve QoL in patients with both solid tumors and hematological malignancies, although there is a lack of awareness of exercise recommendations, timing, and referral to such programs. New evidence indicates that physical activities improve CRF, which can lead to a reduction in CV mortality. Furthermore, cardiac dysfunction is a side effect of many oncological treatments, which may be mitigated by exercise interventions according to preclinical studies and recent publications. Nevertheless, specific physical exercise programs are not widely used in cancer patients. Thus, the goal of this review was to describe the current evidence on the benefits of exercise in cancer patients, the gaps that remain, and an approach to exercise prescription.

## 1. Introduction

The effects of cancer and its treatments including chemotherapy, radiation, hormonal, and/or biological therapies can affect cardiovascular (CV) health, causing worsening of the CV profile, prognosis, cardiac function, cachexia, fatigue, and quality of life (QoL). CV mortality is over 20% in patients with breast, endometrial, and thyroid cancers and is almost 30% in those with prostate cancer [1, 2]. Over half of cancer patients report moderate-to-severe fatigue during treatment, of which 25% have persistent fatigue more than 5 years after treatment completion, and 5–26% of cases have a decline in cardiorespiratory fitness (CRF) [3, 4]. Moreover, many current cancer therapies cause significant adverse CV events, such as decreased cardiac function or heart failure. Furthermore, CV disease (CVD) and cancer share risk factors such as obesity, hyperglycemia, hypertension, and hypertriglyceridemia-induced inflammation, promoting carcinogenesis and tumor progression [5, 6].

Physical exercise has been positioned as a strategy to improve different aspects of life of cancer patients (Table 1, Ref. [7, 8]), and is recommended as a therapy in cardio-oncology guidelines. Different studies have shown the benefits of exercise in decreasing CV mortality, and improving CRF and QoL. Accurate exercise prescription is mandatory to obtain the expected benefits of reducing CV risk and mortality [7, 8].

**Table 1.** **Difficulties and benefits of exercise prescription in cancer patients**.

Difficulties [7, 8]	Benefits [7, 8]
Limited access to resources to support exercise	Improves cardiorespiratory fitness
Limited financial coverage	Ameliorates quality of life
Difficulty of adherence and motivation	Reduces fatigue
	Improves the cardiovascular profile
	Reduces cardiovascular mortality

Here, we provide an overview of the current evidence on the benefits of exercise interventions in cancer patients in terms of mortality and CRF, primary prevention of cardiotoxicity, fatigue, and QoL as well as the optimal timing of physical exercise and prescription recommendations.

## 2. Cancer and CV Health

Cancer survivors have an increased risk of long-term CV mortality compared to the general population, either because of unhealthy lifestyles or the toxicities of their treatment. The risk of death from CVD differs depending on the type of cancer and is estimated to be between 3% and 5% for brain and liver cancers, but can increase to 30–40% for prostate and bladder malignancies [1, 9, 10, 11]. Furthermore, significant and marked impairment of CRF has been demonstrated along the entire disease continuum, which may not recover after the completion of treatment. An inversely proportional correlation between CRF and CV mortality has been described, leading to a significant increase in mortality risk with decreasing CRF levels. This relationship is independent of traditional CV risk factors [4, 12, 13, 14, 15].

Cachexia is a severe complication of cancer that negatively affects QoL, response to chemotherapy, and survival; it affects more than 50% of cancer patients and accounts for up to 20% of cancer-related deaths. Cachexia is considered a multi-organ disease that involves different tissues and organs, including the heart. Cardiac cachexia has been observed in some cancer types, such as lung, pancreatic, and gastrointestinal cancers, and occurs primarily as a consequence of cardiac protein loss [16, 17].

Cancer therapy can also adversely affect cardiac structure and/or function, resulting in ventricular dysfunction that may be asymptomatic or symptomatic, manifesting as heart failure. This condition has been termed “cancer therapy-related cardiac dysfunction” (CTRCD) and occurs with many cancer therapies. The incidences of CTRCD induced by the commonly administered therapies anthracyclines (ACs), human epidermal growth factor receptor 2 (HER2)-targeted agents (e.g., trastuzumab), and immune checkpoint inhibitors are 5–40% (depending on dose and duration of exposure), 18%, and 15% [18, 19, 20, 21], respectively. Moreover, cardiac diastolic function may be impaired even with a low dose of AC as well as with new cancer therapies, which some authors consider a precursor to cardiovascular events [22, 23, 24].

**Fig. 1.**
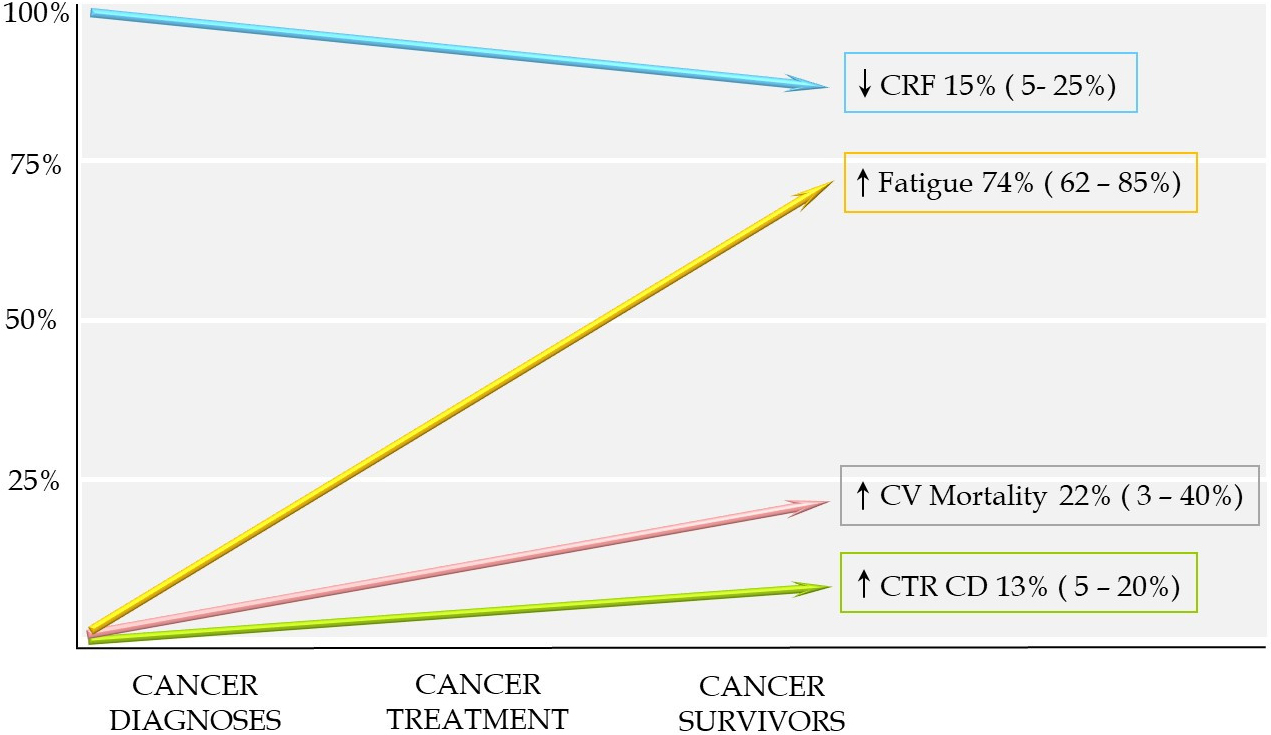
**Effects of cancer in cardiovascular health**. CRF, cardiorespiratory fitness; CTRCD, cancer therapy-related cardiac dysfunction; CV, cardiovascular.

Both cancer survivors and patients undergoing active treatment report fatigue in 62–85% of cases, as well as impairment of health-related QoL (HRQoL) (Fig. 1). The mechanisms underlying cancer-related fatigue suggest the involvement of complex multifactorial processes linked to a range of molecular/physiological processes such as inflammation, as well as the cumulative effects of cancer treatment and psychological factors [25].

## 3. Effects of Exercise on Cancer Patients

### 3.1 Exercise’s Effect on CV Mortality 

In current guidelines, physical exercise has been proposed to reduce morbidity and mortality in cancer patients [7, 8]. Although randomized controlled trials (RCTs) are lacking, current published meta-analyses and retrospective studies provide reliable evidence to support physical exercise as a strategy to decrease CV mortality (Table 2, Ref. [26, 27, 28, 29, 30]). This benefit can be partly explained by the fact that a considerable proportion of patients are at significant risk of CVD at the time of cancer diagnosis or have a worsened CV profile during treatment. In addition, the risk of CV toxicity is higher in patients with hypertension, hyperlipidemia, smoking, diabetes, or obesity [31]. Exposure to chemotherapy leads to vascular endothelial dysfunction, which contributes to the development of CVD. Current evidence shows that exercise training improves vascular endothelial function and wall thickening, especially in breast and prostate cancer survivors [32, 33, 34]. Thus, a supervised and structured physical exercise program would improve the CV profile of cancer patients and, therefore, potentially reduce CV mortality in the medium and long term.

**Table 2.** **Trials evaluating the effect of exercise on mortality and CRF**.

Study	Type	Cancer	N	Results
Kuiper JG *et al*. 2012 [26]	Prospective	Colorectal	1339	PA of ≥18 MET-hours/week had significantly lower colorectal cancer-specific mortality and all-cause mortality
Beasley JM *et al*. 2016 [27]	Prospective	Breast	2265	Reduction in death from any cause and death from breast cancer for women who met the PA of 10 MET-hours/week 18 to 48 months post-diagnosis
Cormie *et al*. 2017 [28]	Meta-analyses	Breast	68,285	Trend to reduced risk of mortality in patients with higher exercise behaviours
	15 cohort studies	Colorectal		
	4 RCT	Prostate		
Scott *et al*. 2018 [30]	Meta-analyses	Breast	3632	${\mathrm{VO}}_{\text{2peak}}$ increased by +2.80 mL $O^{2}$ × ${\mathrm{kg}}^{-1}$ × $\min^{-1}$ with exercise therapy compared with +0.02 mL $O^{2}$ × ${\mathrm{kg}}^{-1}$ × $\min^{-1}$ in the control group
	48 RCT	Prostate	1990 (55%) Ex	
		Lung, Hematologic	1642 (45%) UC	
		Colorectal		
		Gastrointestinal		
Kim *et al*. 2021 [29]	Retrospective	Breast	39,775	All exercise intensities had a lower risk of CVD

CVD, cardiovascular disease; Ex, exercise; PA, physical activity; RCT, randomized control trial; UC, usual care; CRF, cardiorespiratory fitness; ${\mathrm{VO}}_{\text{2peak}}$, oxigen consumption peak; MET, metabolic equivalents task units.

In addition to the positive impacts of physical exercise on the CV profile, the role of muscle-strengthening physical activity is essential for cancer patients and is associated with a lower risk of cancer mortality. This favorable effect could be explained by the reduction in systemic inflammation or insulin resistance [35, 36]. Recently, a J-shaped relationship has been observed between muscle-strengthening activities and cancer mortality, with the greatest reduction in risk occurring with 30–60 min of activity per week [37].

Cancer cachexia negatively affects survival. Exercise, as a therapeutic approach to decrease skeletal muscle degradation and body weight loss, could be adequate therapy. However, cachexia generally appears late in the disease, and there have been very few robust RCTs to determine the best therapeutic approach using physical activity [16].

### 3.2 Exercise’s Effect on CRF

Cancer patients present with significant impairment of CRF, which can be improved by exercise therapy. Scott *et al*. [30] conducted a meta-analysis to evaluate the effects of exercise therapy on adult-onset cancer patients as measured by peak oxygen consumption (${\mathrm{VO}}_{\text{2peak}}$), an integrative assessment of global CV function. A significant increase of ${\mathrm{VO}}_{\text{2peak}}$ in the exercise group compared with usual care was reported, with no significant differences in terms of safety, which favored exercise therapy (Table 2). Furthermore, in breast and prostate cancers, an increase in CRF has been observed in patients involved in physical exercise programs, whereas a decrease has been observed in the control groups [38, 39].

### 3.3 Exercise as the Primary Prevention of CTRCD

Preclinical studies have shown that exercise interventions protect against AC-induced cardiotoxicity in rodents; less doxorubicin accumulation in cardiac tissue may be the key underlying mechanism [40, 41, 42]. Furthermore, an experimental study in rats demonstrated that exercise preserved cardiac function and attenuated the autophagic response in the heart and tumor tissues in cancer-induced cardiac cachexia [43]. In humans, there are only a few studies with a limited sample size that have evaluated the cardioprotective effects of exercise (Table 3, Ref. [44, 45, 46, 47, 48]).

**Table 3.** **Trials evaluating cardioprotection strategy with exercise**.

Study	Type	Cancer	Cancer Therapy	Trial intervention	Exercise specification	N	Primary outcome measures
Haykowsky *et al*. 2009 [47]	Single group study	Breast Cancer HER2 positive	Trastuzumab	Exercise	3 days per week during 4 m of trastuzumab therapy	N = 17	No statistically significant change in LV volumes, mass and ejection fraction
					5 min warm-up		
					30–60 min cycle at a HR equal to 60% to 90% of peak oxygen consumption.		
					5 min cool down		
Kirkham *et al*. 2017 [44]	Two-arm proof-of-concept randomized controlled trial	Breast cancer	Anthracycline	Single bout exercise training vs usual care	Single bout of supervised treadmill exercise:	N = 24	Change in biomarkers
			Doxorubicin		10 min warm-up	11 = Ex	No change in echocardiographyic paramteres
					30 min at 70% of age-predicted HR	13 = UC	
					5 min cool down		
Ma Z *et al*. 2018 [46]	Randomized controlled trial	Breast Cancer	Anthracycline	Exercise training vs usual care	16-week exercise supervised program	N = 64	LVEF significantly increased after chemotheraphy in EX group and decrease in control group, statistically significant (*p *< 0.05)
						33 = EX	
						31 = UC	
Kirkham *et al*. 2020 [45]	Prospective, non-randomized controlled trial	Breast Cancer	Anthracycline	Exercise training vs usual care	3 sessions per week	N = 37	No difference on resting cardiac function
					20–30 min of treadmill, elliptical, or cycle ergometer	26 = EX	
					aerobic exercise at 50–75% of age-predicted HR	11 = UC	
Hojan *et al*. 2020 [48]	Randomized controlled trial	Breast Cancer HER2 positive	Trastuzumab	Exercise training vs usual care	Daily sessions 9 w	N = 47	Statistically significant decrease of the LVEF (*p *< 0.05) in the control group compared to the intervention group
					Endurance:	26 = EX	
					2 min warm-up 45 min aerobic activities	21 = UC	
					3 min relaxation period		
					Strength: 40–45 min		

Min, minutes; EX, exercise; UC, usual care; HR, heart rate; LVEF, left ventricular ejection fraction; HER2, human epidermal growth factor receptor 2; LV, left ventricle.

In patients with breast cancer treated with AC, Kikhram *et al*. [44, 45] found no differences in resting cardiac function when comparing patients who performed aerobic exercise with the control group. However, an improvement in hemodynamic responses was detected by increasing cardiac output and decreasing systemic vascular resistance, which indicated a positive pathophysiological impact on the CV profile. In addition, Ma *et al*. [46] reported a positive result in the prevention of ventricular function decline in the physical exercise group.

Pertaining to preventing CTRCD secondary to trastuzumab therapy, discrepancies are found in the medical literature regarding the potential benefits of exercise in this clinical scenario. A prospective study by Haykowsky *et al*. [47] analyzed HER2-positive breast cancer patients who did aerobic training during the first 4 months of adjuvant trastuzumab, and underwent cardiac magnetic resonances before and after the exercise protocol. Left ventricular cavity dilation and worsening of ejection fraction were observed despite aerobic exercise training. Nonetheless, an RCT conducted by Hojan *et al*. [48] reported a statistically significant decrease of left ventricular ejection fraction (LVEF) in the control group compared to the intervention group.

Concerning diastolic function, a prospective cohort study of HER2-positive breast cancer women receiving AC and trastuzumab reported that physical activity of moderate-to-vigorous intensity was associated with better diastolic parameters (higher E/A and lower E/e’ ratio) [49]. Preclinical evidence and current clinical data available suggest that physical exercise may prevent the decline of LVEF secondary to cardiotoxic treatments, especially in selected patients; however, larger RCTs are needed to clarify the role of physical exercise in preventing cardiotoxicity in cancer patients.

### 3.4 Exercise’s Effects on Fatigue and QoL

There is strong evidence for the beneficial effect of exercise interventions on reducing fatigue and improving HRQoL in both solid tumors and hematological malignancies compared to usual care [50, 51, 52].

In solid tumors, the benefits of exercise have been reported in several cancer types, such as breast, colorectal cancer (CRC), lung, and prostate cancers. A meta-analysis by Juvet *et al*. [53] investigated the effects of exercise interventions on women with breast cancer during and after treatment (chemotherapy and/or radiation) and demonstrated that regular exercise decreases fatigue; this benefit persisted at the 6-month follow-up compared to usual care. Exercise programs in patients with CRC have been shown to improve several patient-reported HRQoL outcomes, including physical function, cancer-specific QoL, sleep quality, and fatigue [54]. This benefit is described in different stages of CRC, either pre-surgery, during chemotherapy, or after treatment. Therefore, exercise after CRC diagnosis is feasible and has beneficial effects on HRQoL irrespective of the timing of chemotherapy or surgery [55]. The role of physical activity in anxiety, depression, sleep quality, and fatigue in lung cancer is also favorable. Moreover, structured exercise with or without nutrition therapy has been shown to reduce cancer-related fatigue and improve QoL in patients with prostate cancer [56, 57].

For hematological malignancies, hematopoietic stem cell transplantation (HSCT) is a standard and curative treatment for some of these illnesses, and more than 50,000 HSCTs are performed annually worldwide. A meta-analysis of 10 RCTs of HSCT has demonstrated that exercise interventions have positive effects on reducing fatigue and improving HRQoL and muscle strength, even if initiated before transplant hospitalization (the concept of “pre-hab”) [58]. Based on that data, Mohananey *et al*. [59] proposed a model of cardiac rehabilitation and exercise in patients undergoing HSCT that included aerobic and strength training.

## 4. Optimal Timing of Physical Exercise and Prescription Approach

### 4.1 Optimal Timing of Physical Exercise

The role of cardiac rehabilitation in cancer patients is well established in current cardio-oncology clinical practice guidelines for pre-, active-, and post-specific treatments [60]. The optimal timing of physical exercise interventions to obtain the expected benefits of physical activity and mitigate chemotherapy-induced side effects is not well defined. However, the current evidence supports the benefit of physical exercise throughout the disease. The concept of “pre-habilitation” has been considered before HSCT and breast cancer treatment, with a significant positive impact observed [57, 61]. Physical exercise to improve HRQoL and diastolic and systolic left ventricular function parameters, reduce fatigue, and increase ${\mathrm{VO}}_{\text{2peak}}$ is also favorable during chemotherapy for breast and colon cancers [49, 62]. Van der Schoot *et al*. [63] investigated the impact of exercise intervention during or after chemotherapy on ${\mathrm{VO}}_{\text{2peak}}$ in the breast, testis, and CRC. Although immediately post-chemotherapy, the decline of ${\mathrm{VO}}_{\text{2peak}}$ was less in the exercise group than during chemotherapy, no differences were found at 1 year post-intervention. Recommendations for physical activity for survivors of breast, CRC, and prostate cancers are unequivocal; however, evidence for long-term gynecologic cancers is limited [64].

### 4.2 Approach to Physical Exercise Prescription 

Accurate exercise prescription is mandatory to obtain the expected benefits of reducing CV risk and mortality, increasing capacity functional respiratory (CFR), and improving psychosocial well-being. Initially, cancer survivors should receive a comprehensive assessment of all components of health-related physical fitness (e.g., CRF, muscle strength and endurance, body composition, and flexibility), with cancer-specific considerations such as the disease stage and toxicities, in order to individualize the exercise prescription. Moreover, during active cancer therapy, an individual’s ability to tolerate exercise may fluctuate from day to day [65].

For the adequate evaluation of these patients, ergospirometry (cardiopulmonary exercise testing) provides an assessment of integrative exercise responses involving pulmonary, CV, neuropsychological, and skeletal muscle systems [66]. It also involves measurements of ${\mathrm{VO}}_{\text{2peak}}$, carbon dioxide production, and ventilation during a symptom-limited exercise test [67].

Global recommendations in cancer patients include aerobic and resistance exercises, warm-up and cool-down activities, and flexibility stretching exercises [8]. An effective exercise prescription includes moderate-intensity aerobic training at least three times per week for at least 30 min (90–150 min per week), for a minimum of 8–12 weeks. Moderate-intensity aerobic training reduces anxiety, depressive symptoms, improves HRQoL, bone health, and sleep in cancer patients. Moreover, it improves the lipid profile, lowers hypertension, and provides CV benefits [68, 69]. Resistance training added to aerobic exercise should be done two times per week, using no less than two sets of 8–15 repetitions with at least 60% of one repetition maximum [7]. However, apart from physical exercise, it is necessary to provide the patient with a comprehensive long-term service including medical evaluation, prescriptive exercise, and modification of cardiac risk factors and education [70].

**Fig. 2.**
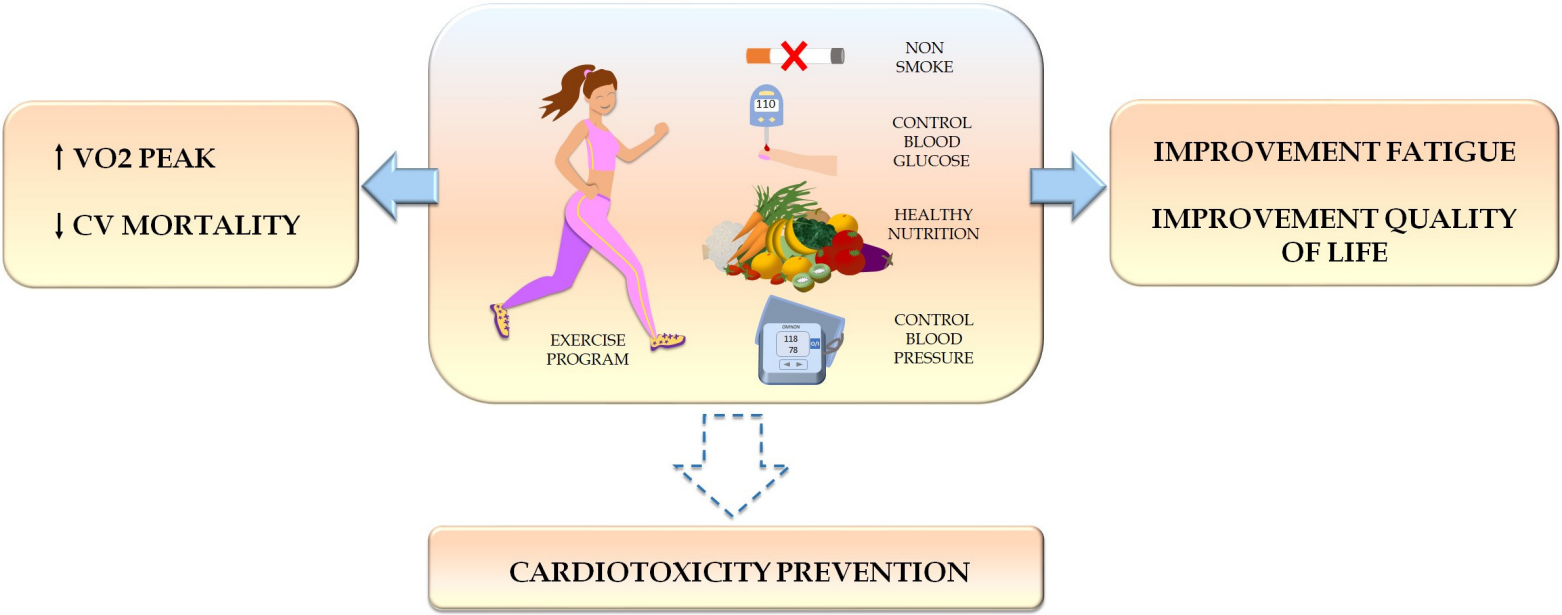
**Benefits of physical exercise in cancer patients**. Physical exercise increases functional capacity, reduces cardiovascular mortality, decreases cancer-related fatigue, and improves quality of life. Current evidence suggests that exercise prevents cancer therapy-related cardiac dysfunction, although more evidence is needed. ${\mathrm{VO}}_{\text{2peak}}$, oxygen consumption peak; CV, cardiovascular.

The goals of cardio-oncology rehabilitation include increasing functional capacity and reducing morbidity/mortality, attenuating the drop in LVEF, decreasing cancer-related fatigue, and improving QoL and psychosocial well-being (Fig. 2). Multidisciplinary teams supported by cardio-oncology units working with different healthcare specialists and aided by advanced cardiac imaging techniques and ergospirometry are required.

## 5. Conclusions

Physical exercise has been demonstrated to provide clinical and emotional benefits to cancer patients. The evidence is reliable for the positive effect of exercise programs in reducing cancer-related fatigue and improving HRQoL, anxiety, depression, and well-being in all cancer populations. The exercise is established as a safe and effective strategy to increase CRF and potentially reduce CV mortality in cancer patients in the medium and long term. Furthermore, preclinical evidence and available clinical data suggest that exercise prevents CTRCD, although more rigorous and extensive RCTs are required, leading to an emerging field of investigation. To obtain the maximum benefits from exercise, this should be individualized according to the patient’s functional capacity, type and stage of cancer, and treatment approach. Moreover, multidisciplinary teams as well as reassessment and continued monitoring of patients during follow-up are critical.
